# Brain age prediction from MRI scans in neurodegenerative diseases

**DOI:** 10.1097/WCO.0000000000001383

**Published:** 2025-05-21

**Authors:** Anthi Papouli, James H. Cole

**Affiliations:** a Hawkes Institute, Department of Computer Science; b Dementia Research Centre, Queen Square Institute of Neurology, University College London, London, UK

**Keywords:** brain age, cognitive decline, machine learning, MRI, neurodegenerative diseases

## Abstract

**Purpose of review:**

This review explores the use of brain age estimation from MRI scans as a biomarker of brain health. With disorders like Alzheimer's and Parkinson's increasing globally, there is an urgent need for early detection tools that can identify at-risk individuals before cognitive symptoms emerge. Brain age offers a noninvasive, quantitative measure of neurobiological ageing, with applications in early diagnosis, disease monitoring, and personalized medicine.

**Recent findings:**

Studies show that individuals with Alzheimer's, mild cognitive impairment (MCI), and Parkinson's have older brain ages than their chronological age. Longitudinal research indicates that brain-predicted age difference (brain-PAD) rises with disease progression and often precedes cognitive decline. Advances in deep learning and multimodal imaging have improved the accuracy and interpretability of brain age predictions. Moreover, socioeconomic disparities and environmental factors significantly affect brain aging, highlighting the need for inclusive models.

**Summary:**

Brain age estimation is a promising biomarker for identify future risk of neurodegenerative disease, monitoring progression, and helping prognosis. Challenges like implementation of standardization, demographic biases, and interpretability remain. Future research should integrate brain age with biomarkers and multimodal imaging to enhance early diagnosis and intervention strategies.

## INTRODUCTION

Ageing has a pronounced impact on the brain and is a major risk factor for poor neurological health. Yet brain ageing affects people differently. Over the last 15 years, the so-called ‘brain-age’ paradigm has emerged as a way of measuring the impact of brain ageing on individuals, by estimating the brain's ‘biological’ age from neuroimaging data, typically magnetic resonance imaging (MRI) scans. The resulting prediction of ‘brain age’ has promise as a biomarker of brain health, that could be used to help better understand and treat neurodegenerative diseases, such as Alzheimer's, Parkinson's or multiple sclerosis (MS). Traditional diagnostic methods often rely on observable symptoms, limiting the potential for early detection. In contrast, brain age provides a noninvasive, quantitative measure that might be sensitive to deviations from healthy brain ageing years before symptoms manifest. This review aims to outline recent developments in the potential applications of brain age, its limitations, and current trends that will shape the future of the paradigm. 

**Box 1:**
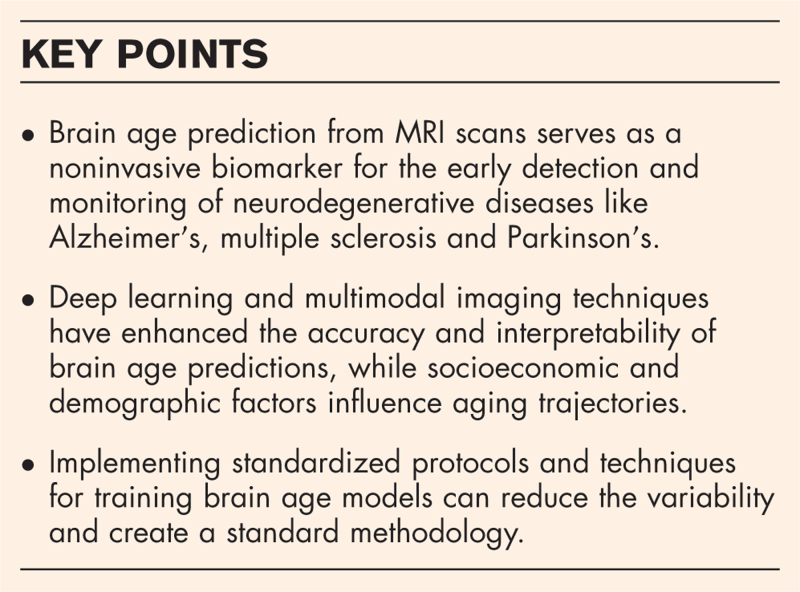
no caption available

## BRAIN AGE, WHAT IS IT AND WHY COULD IT BE USEFUL?

Brain age has its origins in the field of biogerontology, where measures of biological age aim to provide insights into individual differences in the impact of the ageing process. Importantly, biological age measures are proposed to offer more information on the ageing process than chronological age alone. Two people may have the same chronological age but may be experiencing ageing-related changes at different rates, with some people appearing ‘older’ or ‘younger’ for their age. This is true in the brain. Typically, the brain decreases in volume during ageing, though the magnitude of these chances can be larger in people with neurodegenerative diseases, even during the asymptomatic stage.

**FIGURE 1:**
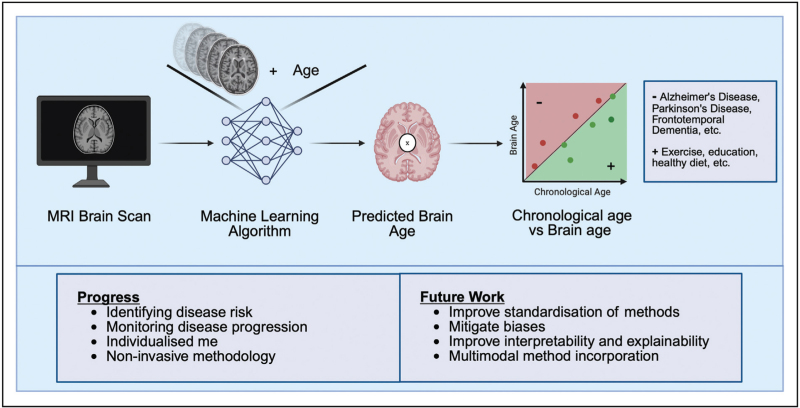
A high-level overview of a Brain Age prediction pipeline. Firstly, an MRI scan of an individual's brain is acquired. Next, the scan is input into a machine-learning model, previously trained on a large number of brain scan from healthy people and their age labels, and outputs the predicted age of the individual, the so-called ‘brain age’. The brain age is then compared to the chronological age of the individual; if the brain age is lower or equal to the chronological age, then the individual is healthy (attributed to high level of education, regular exercise, healthy diet and more). If the brain age is higher than the chronological age, then the individual is likely to have poorer brain health than expected for their age, potentially due to a disease process (for example, Alzheimer's disease, Parkinson's disease, frontotemporal dementia or another neurodegenerative disease).

Brain-age takes advantage of this typical pattern of brain ageing and uses machine learning models trained on large datasets of healthy individuals to predict age from MRI data (see Fig. 1). Once trained, models can predict the ‘brain age’ of new individuals from their brain scan. The difference between an individual's predicted brain age and chronological age is referred to as the brain-age ‘gap’, brain-age ‘delta’, or brain-predicted age difference (brain-PAD) [1]. This can then be used as a biomarker for ageing and neurodegenerative disease research. A positive brain-PAD suggests accelerated brain aging, while a negative brain-PAD may indicate delayed ageing.

Brain age is noninvasive and quantitative, much like other neuroimaging derived phenotypes, such as measures of brain volume, cortical thickness, lesion loads or white-matter tracts. However, brain age differs from these measures in that it is designed to be applied at the individual level, placing a single person's neuroimaging data in the context of a wider population (i.e., the model's training dataset). The individualized nature of the metric makes it suitable for clinical applications, where the goal is often to make decisions regarding individual patients.

## KEY RECENT BRAIN AGE RESEARCH IN NEURODEGENERATIVE DISEASES

The application of brain age in neurodegenerative diseases has yielded a breadth of findings that reinforce its sensitivity to disease risk, cognitive decline, and disease progression. Research consistently shows that individuals with neurodegenerative conditions have a brain-predicted age significantly older than their chronological age, suggesting increased brain ageing [1,2].

However, demonstrating brain-age differences in manifest disease has limited utility. More compellingly, evidence suggests that brain age is altered during the early stages of neurodegeneration. For example, in a group with cognitive impairment though generally without a dementia diagnosis, higher brain-age was associated with CSF amyloid and tau levels and poorer cognitive performance [2]. Furthermore, individuals with Alzheimer's or mild cognitive impairment (MCI) exhibit older brain ages compared to their chronological age due to white matter hyperintensities [3] or structural changes in the fronto-temporo-parietal cortices [4]. These changes often precede cognitive symptoms [5,6]. For example, Liu and colleagues developed a conversion risk estimation system (CRES) using MRI-derived brain age to predict the progression from MCI to Alzheimer's. They found that each additional year of brain-PAD increased the risk of Alzheimer's conversion by 4.6%, even when accounting for Mini-Mental State Examination (MMSE) score [1]. This aligns with Gaser *et al.*'s seminal early brain-age work, where their BrainAGE metric was able to predict conversion to Alzheimer's in people with MCI [7].

Similarly, Antoniades *et al.* explored brain age heterogeneity in relation to cognitive decline and Alzheimer's neuropathology [3]. They found that individuals with advanced structural and functional brain ages exhibited greater neurodegenerative features, including white matter hyperintensities and Alzheimer's-specific atrophy patterns. These structural abnormalities were strongly associated with cognitive deterioration, measured by tools such as the MMSE and progression to MCI.

Beyond Alzheimer's, altered brain age has been observed in other neurodegenerative conditions. In frontotemporal dementia (FTD), region-specific cortical atrophy contributes to accelerated brain ageing [8], while in Parkinson's, brain age correlates with motor symptom severity and cognitive decline [9]. In Huntington's disease, brain-PAD shows potential for stratifying patients according to disease stage, and can differentiate premanifest and symptomatic patients [10]. In MS, brain-PAD is characteristic of accelerated ageing and is associated with changes in physical and cognitive ability [11^▪^]. These findings highlight brain age's role in providing specific insights in different neurodegenerative diseases. Promisingly, brain age has also recently been deployed in a clinical trial of a remyelination therapy [12^▪^]. Compared to placebo, patients with MS who received bexarotene showed a reduced brain-PAD after treatment, and this decrease correlated with measures of remyelinating activity. This highlights the exciting potential that brain age can be therapeutically altered, reducing the gap between brain-age and chronological age, meaning that brain age could be used as an outcome measure in clinical trials of other neuroprotective treatments.

As neurodegenerative diseases progress, brain age scores increase, indicating underlying structural deterioration, supporting its use as a disease progression monitoring tool. For example, Ly and colleagues demonstrated that individuals with MCI had higher rates of increases in brain-PAD over time compared to cognitively unimpaired controls and to people with Alzheimer's [13]. They speculated that the Alzheimer's patients had already undergone a period of accelerated changes (as their brain-PAD was greater than MCI), while brain age measures were capturing active neurodegeneration in the MCI group. This highlights the potential of brain age for tracking disease progression, even in the early stages.

Brain age has also recently been shown to be a predictor of cognitive decline. Cumplido-Mayoral *et al.* showed that higher brain-PAD was linked to declines in attention, executive function, and episodic memory, driven by structural changes in regions like the hippocampus, entorhinal cortex, and amygdala [14]. Importantly, the correlation between higher brain-PAD scores and cognitive decline was still evident in amyloid-negative individuals, indicating sensitivity to early-stage neurodegeneration.

Evidence now suggests that brain age can also reflect both neurodegenerative and systemic aging processes, with Casanova and colleagues reporting associations to conditions like obesity, diabetes, hypertension, and stroke [15^▪▪^]. Supporting this, Marseglia *et al.* found that greater brain-PAD scores were associated with vascular-related conditions, including stroke and diabetes, as well as C-reactive protein and glucose, reflecting inflammation and metabolic alterations [16]. These nonneurodegenerative factors contributed to older-appearing brains and were to physical inactivity and poorer cognitive function. Going further, Casanova *et al.* reported robust associations between higher brain-PAD and mortality, even when looking at people with normal cognition at the time of scanning. This aligns with previous studies, including our own work [17,18]. Given the links between systemic health and neurodegenerative disease risk [19], brain age appears sensitive to both factors. In fact, Moguilner *et al.*'s recent findings suggest that socioeconomic disparities and environmental exposures also contribute to alterations in brain age [20]. There is a complex interplay between socioeconomic factors, systemic conditions and neurological health, and while brain age is not suited to disentangling this interplay, it could be a useful integrative marker of overall brain health.

Intriguingly, Casanova *et al.*'s impressive study also assessed proteomic data, finding 33 out of almost 5000 proteins that were associated with brain-PAD after multiple comparison correction [15^▪▪^]. Some of the identified proteins had previously been linked to biological ageing, cellular senescence and dementia. Meanwhile, Liu and colleagues [21] also took a proteomic approach to brain age, assessing almost 3000 proteins, finding 13 significant associations. Despite using different datasets, different brain-age models, and different proteomic platforms, both Casanova and colleagues and Liu and colleagues found the same ‘top-hit’ protein, *growth differentiation factor 15* (GDF15). This cytokine, involved in macrophage inhibition via transforming growth factor-β (TFG-β) signalling, is worthy of follow-up investigation. Overall, these promising results suggest that brain-age not only reflects neurological health, but also a broader biological health status in ageing, and could be used to identify novel therapeutic pathways as well as identify individual risk of poorer ageing-related outcomes.

Recent advances in deep learning models have significantly improved the accuracy and interpretability of brain age estimation. Convolutional neural networks (CNNs) and other AI models can detect complex neuroanatomical patterns that traditional methods might overlook [22]. Our recent work has even shown how it is possible to train a model to predict age accurately with one MRI modality, then use transfer learning to adapt to a different MRI modality, overcoming the need for large training datasets in new contexts [23]. However, a common critique of these models is their “black-box” nature, where sometimes millions of different parameters are learned and combined in nonlinear fashion, making it difficult to what drive the predictions made [24]. To improve interpretability, researchers have developed anatomically interpretable deep learning frameworks. For example, Yin and colleagues recently introduced a saliency-based deep learning approach that highlights specific brain regions contributing to aging predictions [25]. Such frameworks could lead to important insights into the mechanisms underlying neurodegenerative diseases and are helping to refine the practical utility of predictive tools for early detection or prognosis.

These findings highlight the significant potential of brain age estimation in understanding and managing neurodegenerative diseases. Its ability to predict MCI-to-Alzheimer's progression, to reflect response to remyelination therapy, to integrate brain changes resulting from systemic and socioeconomic factors, and to assess mortality risk gives brain-age real potential as a tool in clinical settings.

## LIMITATIONS OF BRAIN AGE

Despite the promise of brain age for neurodegenerative diseases, several limitations and challenges must be addressed before it can be fully integrated into clinical practice.

A major challenge in brain age is the lack of standardization across models and methodologies. Machine learning algorithms used for brain age prediction are often trained on different datasets with varying neuroimaging protocols, scanner types, and preprocessing techniques, leading to inconsistent predictions and difficulty in comparing findings across studies. For example, differences in MRI acquisition parameters have been shown to influence brain-PAD scores, raising questions about clinical reproducibility [26]. Without standardized methodologies, establishing brain age as a dependable diagnostic tool remains challenging. However, recent efforts by Dular and Špiclin have begun addressing this limitation [27], as highlighted in emerging trends.

Another limitation is that most brain age models are trained on datasets composed largely of participants from North America and Europe, limiting their generalizability to underrepresented populations. Ethnic and demographic biases in training data can lead to inaccurate predictions when these models are applied to diverse groups. For instance, a study assessing brain age in a Latin American cohort found that existing models overestimated brain age compared to models trained on European datasets [20]. This underscores the necessity of developing inclusive brain age models that account for cultural, ethnic, and socioeconomic diversity to ensure accurate predictions across global populations.

## EMERGING TRENDS IN NEUROIMAGING AND HOW THEY CAN ENHANCE BRAIN AGE

As brain age continues to be developed as a potential biomarker for neurodegenerative diseases, several emerging trends in neuroimaging and computational neuroscience are set to enhance its accuracy, interpretability, and clinical applicability. Recent advancements in multimodal neuroimaging, deep learning and standardization of protocols, are transforming brain age from a research tool into a clinically viable metric.

Brain age models have relied primarily on T1-weighted MRI, but newer approaches incorporate diffusion-MRI, T2-FLAIR, functional MRI (fMRI) or electroencephalography (EEG) to provide a more comprehensive assessment of brain health. This multimodal approach captures both structural and functional changes associated with aging and disease, enhancing the ability to distinguish between normal aging and neurodegenerative processes. Recent studies have shown that combining fMRI and EEG improves brain age predictions, revealing how different brain networks contribute to aging at varied rates [20]. For example, Millar and colleagues demonstrated that integrating structural MRI with functional connectivity significantly enhances the accuracy of brain age predictions in individuals with Alzheimer's. Their multimodal models detected early-stage neurodegenerative changes, with functional connectivity capturing distinct aging patterns that structural MRI alone could not identify [2]. This combination offers a nuanced understanding of both normal and pathological aging trajectories.

The application of deep learning models, particularly CNNs, has significantly improved the accuracy of brain age estimation [8,22,26]. With the use of explainable AI techniques like saliency maps, the brain regions most influential in age predictions can be highlighted, improving interpretability. Though ageing impacts the entire brain, key regions associated with ageing include the hippocampus and prefrontal cortex [25]. Advancements in explainable AI could provide new insights into patterns of ageing-related brain atrophy in neurodegenerative diseases like Alzheimer's, Parkinson's and MS.

Addressing the challenge of model variability and lack of standardization, Dular and Špiclin recently introduced the Brain Age Standardized Evaluation (BASE) framework, which establishes standardized protocols for assessing brain age prediction models using T1-weighted MRI data [27]. Their work highlights the critical need for consistency in data preprocessing, model evaluation, and reporting practices. BASE facilitates cross-comparison of different machine learning models by applying uniform testing on multisite datasets with varied MRI acquisition protocols. This framework should enhance the reproducibility and robustness of brain age models, moving the field closer to clinical applicability. Engaging the scientific community in such endeavours will also be essential, which is one of the goals of the ENIGMA consortium's Brain Age working group (https://enigma.ini.usc.edu/ongoing/enigma-brainage). The introduction of such standardized evaluation tools represents a pivotal step toward integrating brain age estimation as a reliable biomarker in both research and clinical settings.

## CONCLUSION

Brain age prediction represents an innovative advancement in neuroimaging, providing a quantifiable and interpretable measure of brain health, that can naturally be applied to individual patients. While limitations exist, recent advancements in deep learning, standardized protocols, multimodal imaging, and demographic inclusivity are refining brain age as a biomarker. Given the increasing prevalence of neurodegenerative diseases, brain age could be incorporated into clinical workflows to aid in early diagnosis, disease monitoring, and even personalized treatment planning.

By using brain age alongside traditional biomarkers, emerging proteomic data, and sophisticated cognitive assessments, clinicians may gain deeper insights into an individual's brain health trajectory, ultimately leading to improved patient outcomes. Future research should focus on implementing standardized methodologies, reducing algorithmic biases, and integrating multimodal data to fully harness the potential of brain age for neurodegenerative diseases.

## Acknowledgements

*None.*

### Financial support and sponsorship

*A.P. is funded by a grant from the Alzheimer's Society (reference 634).*

### Conflicts of interest

*J.C. is a shareholder in and advisor to Brain Key and Claritas HealthTech PTE.*

## REFERENCES AND RECOMMENDED READING

Papers of particular interest, published within the annual period of review, have been highlighted as:

▪ of special interest
▪▪ of outstanding interest
